# The Effects of Mycoplasma Contamination upon the Ability to Form Bioengineered 3D Kidney Cysts

**DOI:** 10.1371/journal.pone.0120097

**Published:** 2015-03-20

**Authors:** Teresa M. DesRochers, Ivana Y. Kuo, Erica P. Kimmerling, Barbara E. Ehrlich, David L. Kaplan

**Affiliations:** 1 Department of Biomedical Engineering, Tufts University, Medford, Massachusetts, United States of America; 2 Departments of Pharmacology and of Cellular and Molecular Physiology, School of Medicine, Yale University, New Haven, Connecticut, United States of America; University of California, Merced, UNITED STATES

## Abstract

Mycoplasma contamination of cell cultures is a pervasive, often undiagnosed and ignored problem in many laboratories that can result in reduced cell proliferation and changes in gene expression. Unless contamination is specifically suspected, it is often undetected in two dimensional (2D) cultures and the resulting effects of mycoplasma contamination are rarely appreciated and can lead to incorrect conclusions. Three dimensional (3D) tissue cultures are increasingly utilized to explore tissue development and phenotype. However, 3D cultures are more complex than 2D cell cultures and require a more controlled cellular environment in order to generate structures necessary to mimic *in vivo* responses and are often maintained for longer time periods. Changes to the microenvironment are assumed to have a more extreme effect upon the success of 3D tissue cultures than 2D cell cultures, but the effects of mycoplasma have not been studied. To test this hypothesis, we grew 2D cell cultures and 3D tissues from pig kidney epithelial cells (LLC-PK1) that were contaminated with mycoplasma and the same stock of cells after mycoplasma removal. We did not observe an effect of mycoplasma contamination on proliferation in 2D monolayer cell culture. However, cyst formation in 3D tissues was altered, with effects upon the number, size and structure of cysts formed. These data serve to reinforce the necessity of testing cell stocks for mycoplasma contamination.

## Introduction

Mycoplasmas are a group of microorganisms that lack a rigid cell wall and their small size (0.3 to 0.8 μm) and the flexibility afforded by the lack of a cell wall allows these contaminating organisms to pass through most sterile filtration units utilized in conventional cell biology laboratories [1]. Mycoplasmas are also difficult to detect because they are not visible under conventional phase contrast microscopy and have a very slow growth rate. Once mycoplasma infects a mammalian cell culture, they can have an impact upon almost all cellular processes including metabolism, proliferation, gene expression, and signal transduction [1–3]. They have been shown to result in artificial apoptosis detection when using DNA fragmentation as the assay [4], can cause inaccurate detection of protein expression or degradation [3, 5], and can impact siRNA stability [6]. Undetected contamination of cell lines with these microorganisms remains a large problem in scientific laboratory environments with several studies showing infection rates of cell lines at an estimated 15–35% but some as high as 60–85%, most likely through poor cell culture techniques [1, 7, 8].

Bioengineered 3D tissues are most often formed from cells originally cultured in 2D monolayer systems and many current 3D systems rely upon established, continuous culture cell lines. These 3D tissues are more complex than 2D monolayers and their success as models of disease, including tumor and cyst models, or of organ development, is measured by their ability to recapitulate *in vivo* systems [9–13]. 3D tissues require a highly regulated and reproducible environment to maintain cellular responses to both extracellular and intercellular cues. Additionally, 3D tissues are grown for longer periods of time, of up to weeks and months, compared to 2D cultures [11, 12]. These longer time periods provide uninterrupted growth for contaminants to become established and increase the likelihood that contaminants can impact cellular function.

Therefore, it is possible that even if mycoplasma contamination does not exert a detectable response in 2D, it will have a significant impact upon the successful growth of the same cells in 3D culture. To test this hypothesis, we have examined the growth of a group of pig kidney epithelial cells (LLC-PK1) cells with different gene knockdowns of two different calcium channels, an isoform of the intracellular calcium channel inositol triphosphate receptor type 3 (InsP3R3) or polycystin 2 (PC2) in both 2D and 3D cultures both before and after mycoplasma removal. We chose to specifically look at PC2 and the InsP3R as mutations to PC2 are associated with polycystic kidney disease, and alterations in calcium signaling via the InsP3R has been implicated as a possible mechanism of cyst development [14–18]. Here, we show for the first time that even though a mycoplasma contamination did not result in a proliferation defect or other notable cell culture defects in short-term 2D culture, the presence of the mycoplasma did have a significant impact upon 3D tissue morphology.

## Results and Discussion

**Fig 1:**
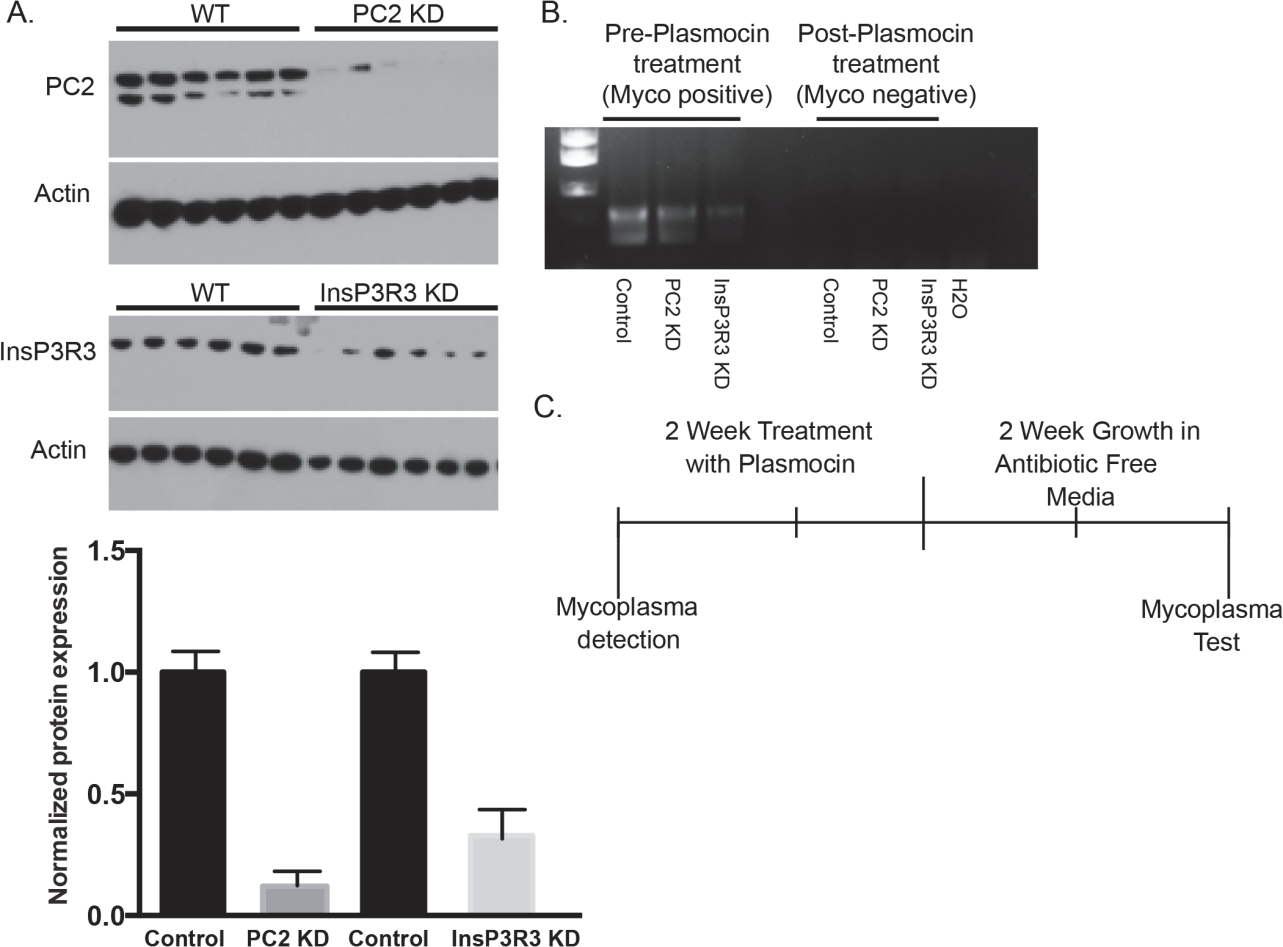
Mycoplasma detection and removal. (A) Top: Representative Western blot of InsP3R3 and PC2 expression after shRNA knockdown. Lanes to the left represent control; lanes to the right represent the knockdown condition. Actin was used as a loading control. Each lane represents a separate sample. Bottom: Quantified analysis of Western Blot. (B) Mycoplasma contamination was detected by PCR before (left lanes), but not after (right lanes) Plasmocin treatment. (C) Mycoplasma was removed from cell lines over 2 weeks by treatment with Plasmocin every other day. Following treatment, the cells lines were grown in antibiotic free media for an additional 2 weeks to promote the growth of any remaining mycoplasma.

**Fig 2:**
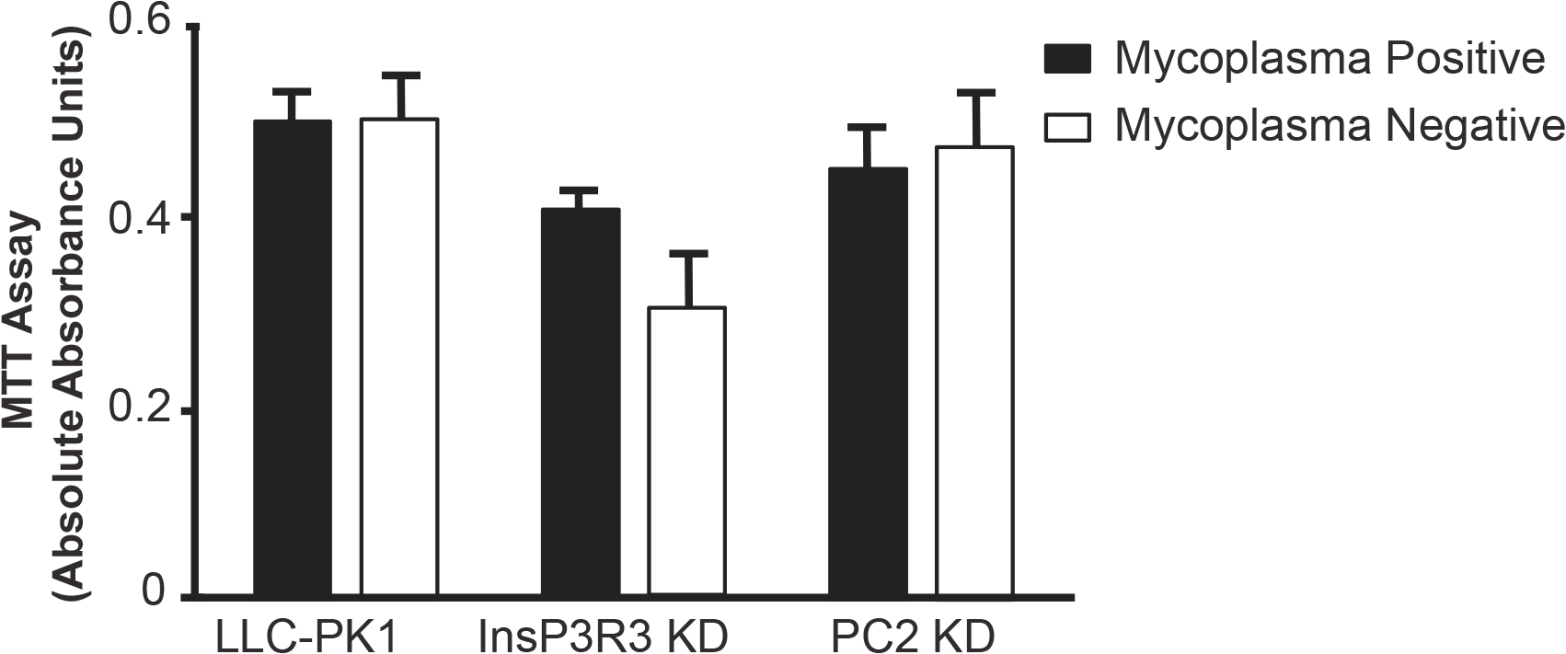
Cell proliferation in 2D cell cultures. Mycoplasma positive and mycoplasma-negative cells were grown for 48 hours and assayed for cell proliferation with an MTT assay. Data represents an average of 8 replicates and the error bars represent the standard error of the mean. The respective p values are: LLC-PK1 = 0.996, InsP3R3 KD = 0.137, and PC2 KD = 0.76.

To examine mycoplasma contamination in 2D culture, we used a group of mycoplasma infected LLC-PK1 cells that had been unmodified or modified with short hairpin RNA (shRNA) to knockdown two different calcium channel proteins. We utilized cells with knockdown of either PC2 (PC2 KD) or InsP3R3 (InsP3R3 KD) with specific shRNAs designed against either PC2 or InsP3R3 [17]. The degree of knockdown was assessed by Western Blot analysis (Fig. 1A), and found to be greater than 70%. Initial mycoplasma contamination (mycoplasma-positive samples) was detected with PCR (Fig. 1B, left lanes) and a mycoplasma detection kit. Testing with the mycoplasma detection kit resulted in a range of readings between 0.9 and 1.68 with readings over 0.1 indicating positive mycoplasma contamination. To remove the mycoplasma contamination, the cells were treated every other day for 2 weeks with Plasmocin and then grown in antibiotic-free media for an additional 2 weeks (Fig. 1C) to confirm the absence of mycoplasma contamination (Fig. 1B, right lanes, mycoplasma-negative samples) [19]. Both the mycoplasma-positive and mycoplasma-negative cell lines were then grown in 2D culture for 48 hours and their proliferation was measured by an MTT assay. Although mycoplasma has been previously reported to alter cell proliferation, the mycoplasma contamination had no significant impact upon the proliferation of the cell lines in 2D culture over the time frame tested (Fig. 2). Moreover, the effects of cell proliferation were not affected by knockdown of either PC2 or InsP3R3. The degree of knockdown was not noticeably different before and after Plasmocin treatment, with the degree of knockdown greater than 70% (compare Fig. 1A with previously published results [17].

Although the effects of cell proliferation over 48 h were not significantly different between the mycoplasma-contaminated or the mycoplasma-negative cells, we extended the study to long-term 3D culture. To examine mycoplasma contamination in 3D tissues, the LLC-PK1 and InsP3R3 knockdown cell lines were grown as 3D tissues as both mycoplasma-positive and mycoplasma-negative cultures. We took particular care in examining the morphology, number and cellular structure of the cells in 3D culture.

**Fig 3:**
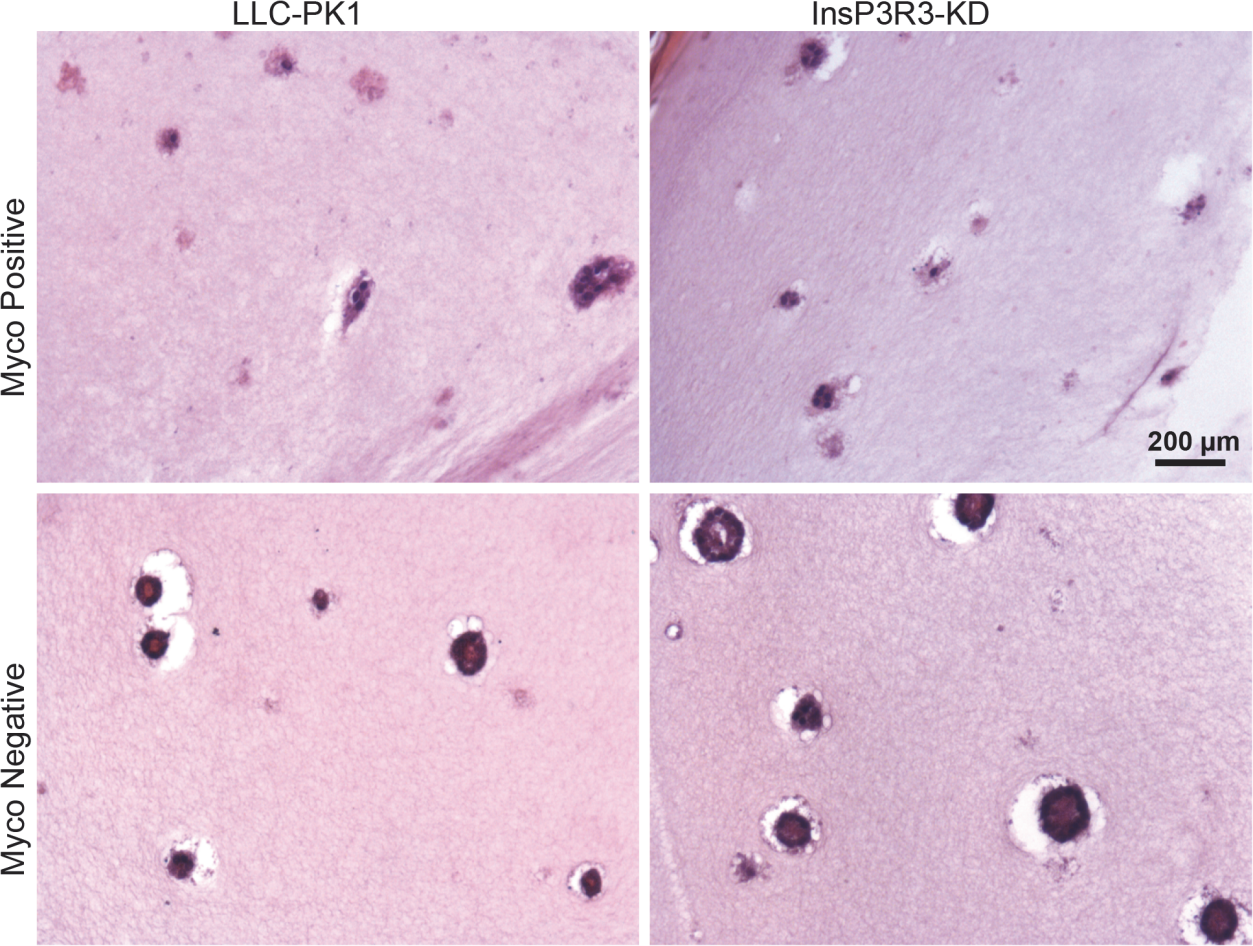
Cyst formation is improved in 3D tissues without mycoplasma contamination. Mycoplasma positive and mycoplasma-negative cells were grown in 3D tissues for 2 weeks at which time the tissues were fixed, sectioned, and stained for H&E. Note that the cysts are more diffuse in the mycoplasma-positive tissues. Scale bars = 200 μm.

**Fig 4:**
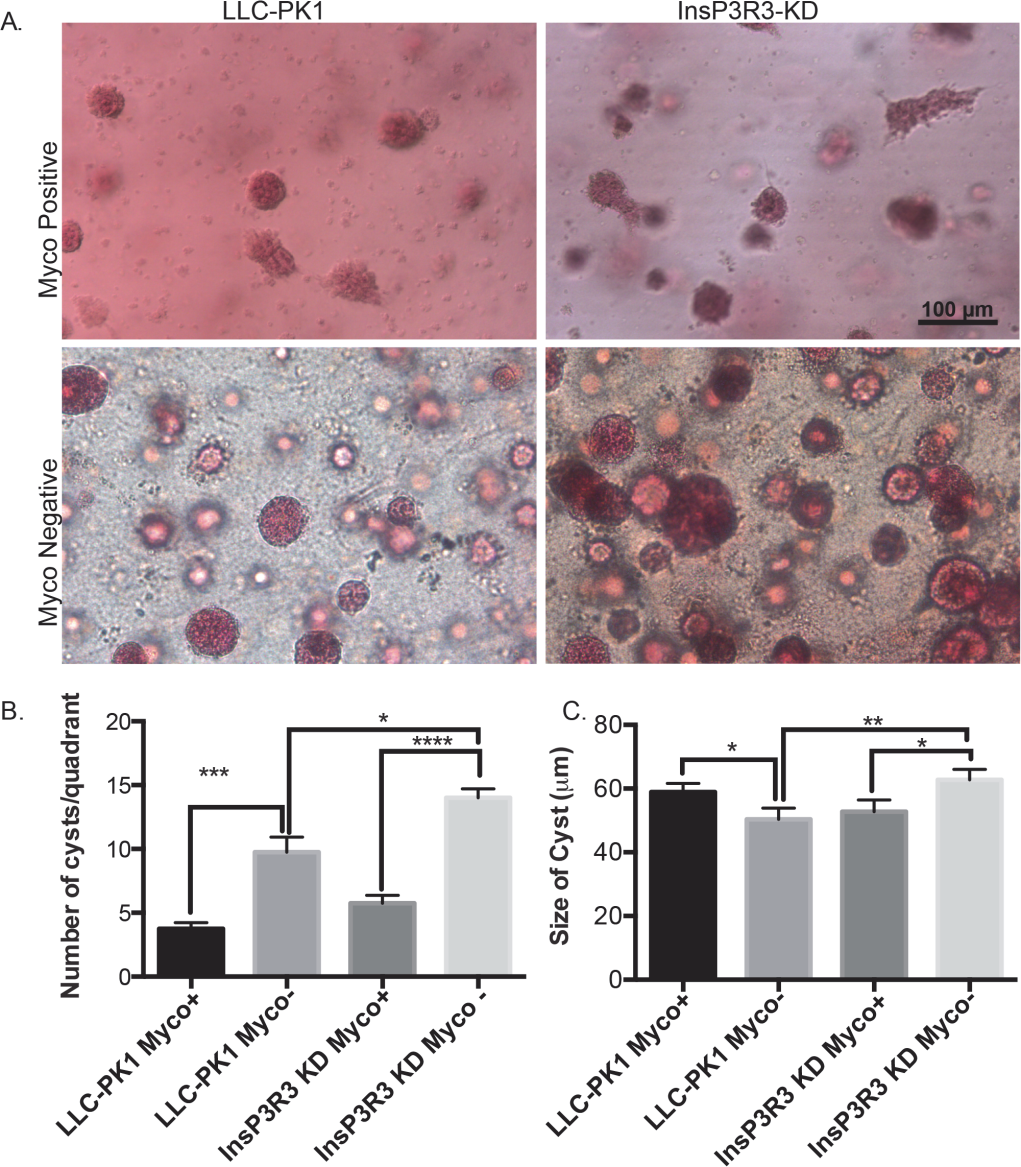
Cyst number is increased in 3D tissues without mycoplasma contamination. (A) Mycoplasma positive and mycoplasma-negative cells were grown in 3D hydrogels for 2 weeks at which time the tissues were fixed and stained with Carmine for whole mount imaging. Note that cysts are more numerous in the mycoplasma negative tissues. Scale bars = 100 μm. (B) Quantified number of cysts across 4 different quadrants. (C) The diameter of the cysts is altered upon treatment of mycoplasma contamination. Data represents the average diameter and the error bars represent the standard error of the mean. * represents p>0.05, ** p>0.01, ***p>0.001, ****p>0.0001

The morphology of multiple regions of numerous 3D tissues was examined after 2 weeks in culture by both hematoxylin and eosin (H&E) (Fig. 3) and whole mount staining (Fig. 4A). H&E staining provided an insight into the organization of the formed cysts including the alignment of cells within the cysts and the hollow structure formed by the cysts. The mycoplasma-positive tissues appeared diffuse and lacked a solid structure compared to the mycoplasma-negative; as examples, the results from LLC-PK1 control and InsP3R3 KD cells are depicted (Fig. 3). Whole mount imaging provided a view of the total number of cysts formed within the tissues (Fig. 4A), with fewer cysts being present in the mycoplasma-positive conditions (as expanded below).

**Fig 5:**
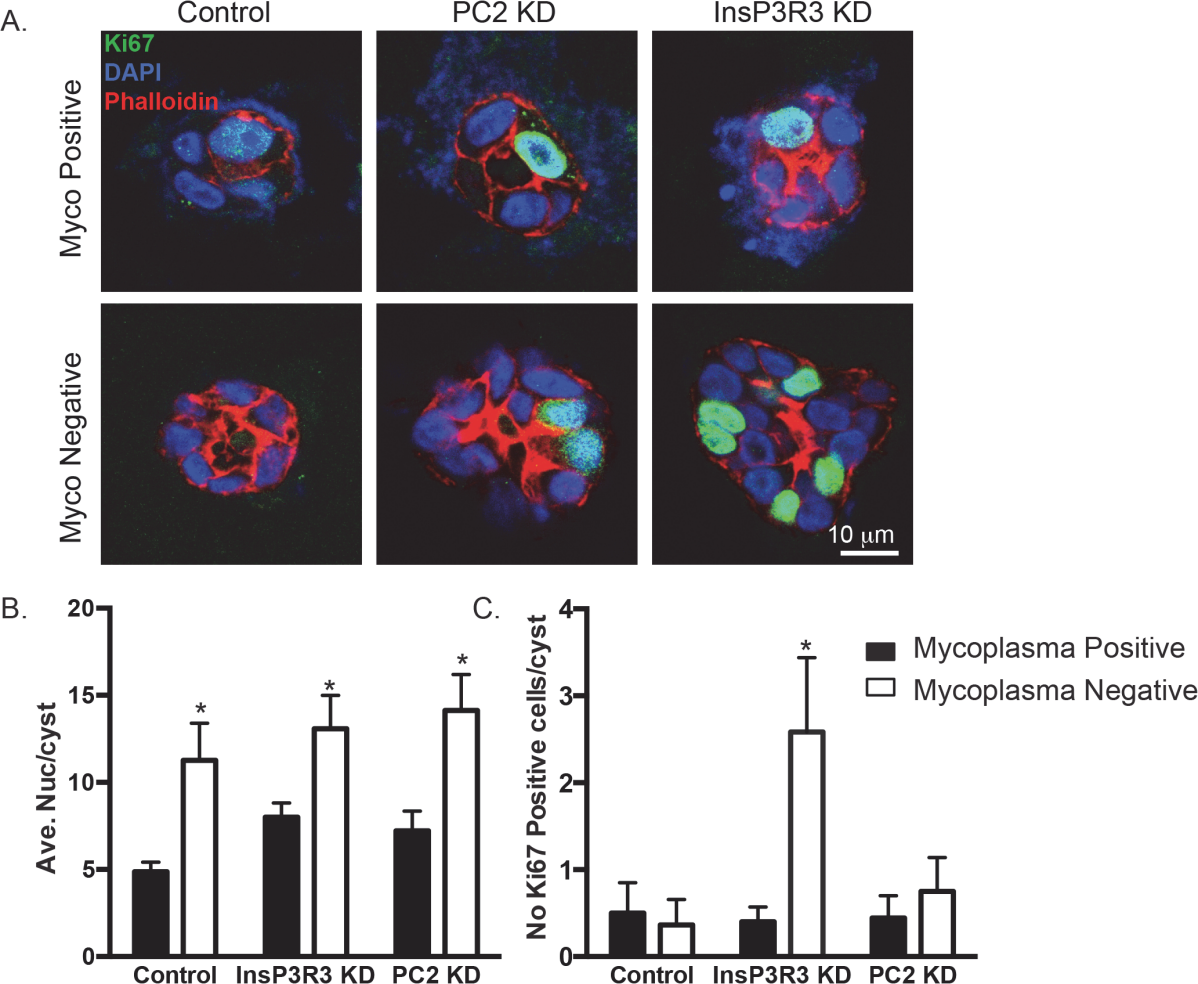
Mycoplasma-positive cysts have fewer proliferative cells. (A). Cysts after 2 weeks in 3D cell culture were stained for an antibody against Ki-67, a marker of cell proliferation (green). Sections were counterstained with actin (using phalloidin red) and DAPI (to denote nuclei, blue). (B) Analysis of the number of cells (as assess by total number of nuclei) per cyst in a cross-sectional area. Analysis is the average of at least 2 different images from three different cyst preparations for each condition, and represents between 9–12 cysts. (C) Absolute number of Ki-67 cells per cyst. Analyses in B and C are the average of at least 2 images from three different cyst preparations for each condition, and represents between 9–12 cysts.

**Fig 6:**
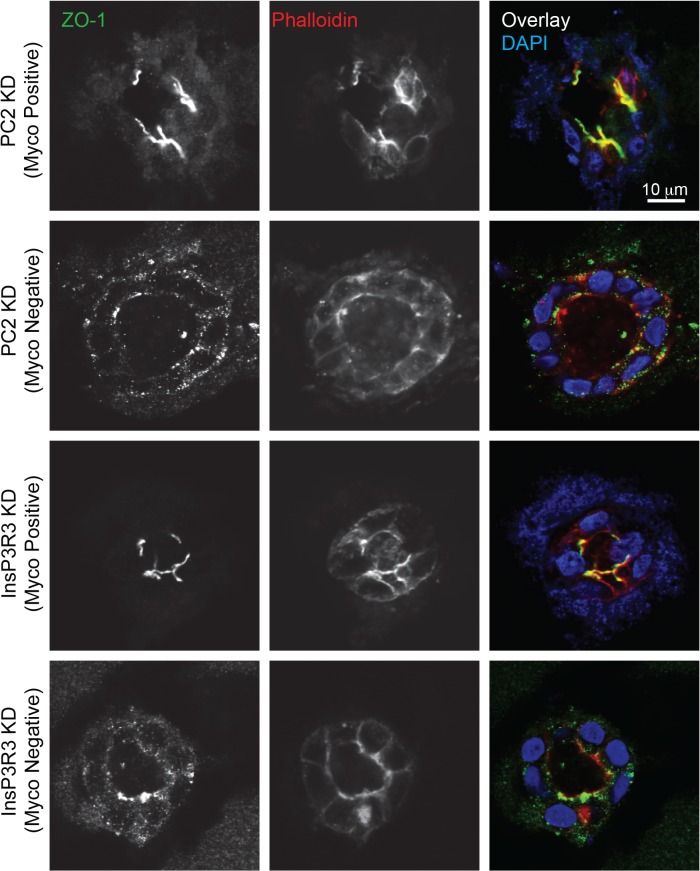
Mycoplasma positive cysts lack organized tight junctions. Cysts were stained for the tight junctional protein, ZO-1 (green), actin (using phalloidin, red), and nuclei (blue) after 2 weeks of 3D culture. Note that ZO-1 expression is reduced and actin is unorganized in mycoplasma positive cultures. Images are representative of at least two different at least 2 images from three different cyst preparations for each condition. Scale bars = 10 μm.

Irrespective of whether the LLC-PK1 cells contained shRNA, the mycoplasma-positive 3D cultures produced significantly fewer cysts than their respective mycoplasma-negative culture counterpart (Fig. 4B). Moreover, there were significantly more cysts with InsP3R3 KD compared with the mycoplasma-negative LLC-PK1 control cysts. Although the results for the PC2 KD cells are not shown, subsequent analysis of cryo-sections (see Figs. 5 and 6) did not show discernable differences between the mycoplasma-positive and mycoplasma-negative conditions.

After 2 weeks of culture, the size of the cysts was not different among the mycoplasma-positive cysts, however, upon treatment with Plasmocin, there was a significant change in the size of the LLC-PK1 cells and the InsP3R3 KD cells (Fig. 4C). These results are consistent with our previously obtained results, where we found that interruption of calcium signaling results in increased cyst development [17]. Interestingly, the overall size of the LLC-PK1 cysts decreased upon mycoplasma treatment, perhaps indicating that the presence of the mycoplasma increased cell proliferation in the LLC-PK1 cells. In contrast, the size of the InsP3R3 KD cysts increased significantly with mycoplasma treatment, suggesting that there was an opposite effect of mycoplasma on the cells with InsP3R3 KD compared with the LLC-PK1 cells. These data suggest that the presence of the mycoplasma occludes the disparity in the cyst size between the InsP3R3 KD and the LLC-PK1 control cysts, reinforcing the idea that mycoplasma contamination has a measurable and important effect on cell growth and 3D cultures.

Although our analysis of cell proliferation in short term 2D cell culture revealed no difference in proliferation, we hypothesized that this parameter would be altered when comparing cells maintained in long term 3D culture conditions. To test if mycoplasma had an effect on cell proliferation, we stained cryo-sections of cysts using an antibody against Ki-67 (Fig. 5A). Analysis of the total number of cells in any given cross section of a cyst showed that there was only a significantly higher number of cells in PC2 KD mycoplasma-negative cells compared to PC2 KD mycoplasma-positive cells (Fig. 5B). We also quantified the total number of Ki-67 positive cells per cyst and found that there was no change in the number of Ki-67 positive cells in the control or the PC2 KD cells between mycoplasma-positive and mycoplasma-negative conditions. However, the InsP3R3 KD cells had a significantly higher number of Ki-67 positive cells in the mycoplasma-negative condition compared to the mycoplasma-positive condition. Taken together, these results demonstrate that mycoplasma only had an effect on cell proliferation in 3D cell culture in InsP3R3 KD conditions, and had minimal effects under 2D cell culture conditions.

Finally, mycoplasma contamination has been shown to affect tight junction formation of canine kidney epithelial cells (MDCK) by depleting arginine in the media [2]. This depletion causes the activation of signaling pathways that lead to a decrease in the rate of the tight junction *zonula occludens* protein-1 (ZO-1) production thus resulting in slower tight junction formation between cells. Tight junctions are a hallmark of epithelial junctions that enable epithelial sheets to compartmentalize components [20]. We therefore examined ZO-1 expression and localization (Fig. 6), and found that the mycoplasma-positive PC2 KD and InsP3R3 KD cysts had less ZO-1 expression around the boarders of the cells compared to the mycoplasma-negative cells. The formation of tight junctions also relies upon an organized actin cytoskeleton. Staining of the actin cytoskeleton using phalloidin revealed a lack of organized actin in the mycoplasma-positive PC2 KD and InsP3R3 KD cysts (Figs. 5A and 6). These results may account for the appearance of more diffuse cysts in the mycoplasma-positive cells in 3D tissues (Fig. 3).

Mycoplasma contamination has been shown to have a number of effects upon cells grown in 2D culture [1, 3, 5]. Here we show that mycoplasma contamination did not have an effect upon cell proliferation as measured by MTT assay in 2D culture, which may reflect the subtle effects of mycoplasma on this cell line. However, no studies have explored the effects of mycoplasma on 3D culture. We find that mycoplasma did have an impact on 3D tissue phenotype after only 2 weeks of cell culture. Specifically, mycoplasma contamination caused a decrease in the number of cysts formed in 3D systems and altered the structure of the cysts that formed in these long-term cultures. These changes are probably related to the dual factors of cell proliferation and cell-cell junctions. Moreover, the effects of mycoplasma are exacerbated depending upon the target and efficiency of shRNA knockdown.

In conclusion, we show that even though mycoplasma contamination may not appear to have a large impact upon cell proliferation in short-term 2D cultures, it can have an impact upon proliferation, cyst size and cellular organization in long-term 3D tissues, where studies are often conducted over several weeks to months. Taken together, our data further indicates the importance of examining established cell lines for mycoplasma contamination as the presence of mycoplasma contamination can dramatically alter both the results and interpretation.

## Materials and Methods

### Cell culture and mycoplasma detection and removal

LLC-PK1 (from ATCC CL-101) cell lines were grown in 2D cell culture in DMEM/F12, 2% (v/v) fetal bovine serum, 0.1% (w/v) hydrocortisone, 1% (v/v) insulin-transferrin-selenium, 0.7% (w/v) 3,3′,5-Triiodo-L-thyronine sodium salt, and 0.02% (w/v) epidermal growth factor in a humidified CO_2_ incubator, 37°C supplied with 5% CO_2_.

Mycoplasma contamination was removed with Plasmocin dosed at 25 μg/mL every other day for 2 weeks. Contamination with mycoplasma was examined by both PCR and with the MycoProbe Mycoplasma Detection kit from R&D Systems two weeks after the last dosing with Plasmocin. The PCR primers were designed with reference to previous publications [7]. The primers used were (5’ to 3’): Forward primer: gtggggagcaaataggattaga and Reverse primer: ggcatgtgatttgacgtctt. The genomic DNA was extracted by lysing the cells in NaOH at 95°C. The 25 μl reaction consisted of: 10 mM dNTP, 500 nM both forwards and reverse primer, PCR reaction buffer, and 1 U Hot-Star Taq. The PCR reaction was conducted on a Applied BioSystems PCR analyzer with a initial 15 minute denaturing step followed by 35 cycles of 95°C for 30 s, 55°C for 30 sec and 72°C for 30 s, followed by a single step at 72°C at 2 min, before holding the reaction at 4°C. Half the reaction volume was then run on a 1.2% agarose gel, pre-stained with ethidium bromide. The gel was imaged using a UV imager and VisionWorks LS software. Both the cell-culture media and cell lysates were used as starting material for PCR analysis. Mycoplasma-positive cells were kept isolated from all other cultures and were never grown in the presence of mycoplasma-negative cultures to avoid cross-contamination.

### Cell proliferation

For 2D cell proliferation assays, 5000 cells were seeded per well of a 96-well plate with n = 8 replicates and allowed to grow for 48 hours. At that time, the media was replaced with fresh media containing MTT reagent (Invitrogen). After a 4 hr incubation, cells were lysed with DMSO and the absorbance was read at 540 nm. Data reflects absolute absorbance values after blank values have been subtracted.

### 3D tissue formation and histology

3D tissues were formed as previously described [11, 17]. Briefly, cells were embedded in a 1:1 mixture of Matrigel and rat tail collagen type I in transwell inserts. For histology, tissues were placed in 10% neutral buffered formalin for 48 hr prior to whole mount staining with Carmine. After whole mount images were taken, tissues were processed further and embedded in paraffin for sectioning and H&E.

For immunofluorescence studies, tissues were placed in 2 M sucrose over night at 4°C and then embedded in frozen tissue optimal cutting temperature matrix (OCT) and stored at −20°C. 8 μm cryosections were fixed in 2% (w/v) paraformaldehyde for 10 min. After blocking in 2% (w/v) bovine serum albumin and 0.2% (v/v) Triton X-100, sections were incubated in the same solution with an antibody against ZO-1 or Ki-67 (BD Pharmingen) overnight at 4°C, and then incubated in AlexaFluor 488 (Life Technologies). Sections were also incubated in phalloidin conjugated to AlexaFluor 548 (Life Technologies) to stain for actin before mounting with anti-fade buffered glycerol with DAPI (Life Technologies). Slides were imaged with a Zeiss 710Duo Confocal microscope (Heidelberg, Germany).

### Statistical Tests and Analysis

Data were analysed using PRISM version 6 (Graphpad). Statistical analyses, with Student’s t-test, or 1-way or 2-way ANOVA followed by Tukey test for multiple comparisons were used. Statistical significance was determined when p-value < 0.05.
